# Occurrence of COVID-19 in cystic fibrosis patients: a review

**DOI:** 10.3389/fmicb.2024.1356926

**Published:** 2024-04-17

**Authors:** Fatemeh Sadat Abolhasani, Masood Moein, Niloofar Rezaie, Parimah Sheikhimehrabadi, Morvarid Shafiei, Hamed Afkhami, Mohammadreza Modaresi

**Affiliations:** ^1^Department of Pathobiology, School of Public Health, Tehran University of Medical Sciences, Tehran, Iran; ^2^Department of Bacteriology and Virology, School of Medicine, Shiraz University of Medical Sciences, Shiraz, Iran; ^3^Department of Bacteriology, Pasteur Institute of Iran, Tehran, Iran; ^4^Department of Microbiology, Islamic Azad University, Arak, Iran; ^5^Nervous System Stem Cells Research Center, Semnan University of Medical Sciences, Semnan, Iran; ^6^Department of Medical Microbiology, School of Medicine, Shahed University, Tehran, Iran; ^7^Pediatric Pulmonary Disease and Sleep Medicine Research Center, Pediatric Center of Excellence, Children's Medical Center, Tehran, Iran; ^8^Cystic Fibrosis Research Center, Iran CF Foundation (ICFF), Tehran, Iran

**Keywords:** cystic fibrosis (CF), SARS-CoV-2, *Pseudomonas aeruginosa*, immune system, respiratory virus, pathogenesis

## Abstract

Cystic fibrosis (CF) is a genetic ailment caused by mutations in the cystic fibrosis transmembrane conductance regulator (CFTR) gene. This autosomal recessive disorder is characterized by diverse pathobiological abnormalities, such as the disorder of CFTR channels in mucosal surfaces, caused by inadequate clearance of mucus and sputum, in addition to the malfunctioning of mucous organs. However, the primary motive of mortality in CF patients is pulmonary failure, which is attributed to the colonization of opportunistic microorganisms, formation of resistant biofilms, and a subsequent decline in lung characteristics. In December 2019, the World Health Organization (WHO) declared the outbreak of the radical coronavirus disease 2019 (COVID-19) as a worldwide public health crisis, which unexpectedly spread not only within China but also globally. Given that the respiration system is the primary target of the COVID-19 virus, it is crucial to investigate the impact of COVID-19 on the pathogenesis and mortality of CF patients, mainly in the context of acute respiratory distress syndrome (ARDS). Therefore, the goal of this review is to comprehensively review the present literature on the relationship between cystic fibrosis, COVID-19 contamination, and development of ARDS. Several investigations performed during the early stages of the virus outbreak have discovered unexpected findings regarding the occurrence and effectiveness of COVID-19 in individuals with CF. Contrary to initial expectancies, the rate of infection and the effectiveness of the virus in CF patients are lower than those in the overall population. This finding may be attributed to different factors, including the presence of thick mucus, social avoidance, using remedies that include azithromycin, the fairly younger age of CF patients, decreased presence of ACE-2 receptors, and the effect of CFTR channel disorder on the replication cycle and infectivity of the virus. However, it is important to notice that certain situations, which include undergoing a transplant, can also doubtlessly boost the susceptibility of CF patients to COVID-19. Furthermore, with an increase in age in CF patients, it is vital to take into account the prevalence of the SARS-CoV-2 virus in this population. Therefore, ordinary surveillance of CF patients is vital to evaluate and save the population from the capability of transmission of the virus given the various factors that contribute to the spread of the SARS-CoV-2 outbreak in this precise organization.

## Introduction

Cystic fibrosis (CF) is a genetic ailment resulting from an autosomal recessive trait. In the United States of America, the estimated prevalence of CF disease is 0.797 per 10,000 individuals; at the same time, among Caucasians who are born alive, the superiority is 1 in 2,500 (Stern, 1997). An association related to CF has expressed concerns about the potential impact of COVID-19 on CF patients, as previous viral infections have been associated with severe outcomes and consequences. Furthermore, a significant number of CF patients already have impaired lung function (Lambrecht et al., 2001). In the United States of America, the estimated prevalence of CF disease is 0.797 per 10,000 individuals, while in Caucasians who are born alive, the prevalence is 1 in 2,500 (Stern, 1997). The CF association is expressing significant concerns about the potential impact of the coronavirus disease on individuals with CF, as previous viral infections have been linked to more severe outcomes. Additionally, a significant proportion of CF patients already have impaired lung function (Lambrecht et al., 2001).

## Etiopathology of cystic fibrosis (CF)

The prevalence of CF has increased due to a genetic mutation within a specific gene located on chromosome 7. This gene encodes a protein referred to as the cystic fibrosis transmembrane conductance regulator (CFTR), which consists of 1,480 amino acids. The CFTR protein has a vital function in regulating the movement of electrolytes at some point of epithelial-cell membranes, and it is also believed to have an effect on intracellular membranes (Stern, 1997; Terlizzi et al., 2022a,b).

One of the foremost complications related to CF is continuous pulmonary infections, which might be the leading cause of respiratory failure in individuals with CF (Meng et al., 2020). A study carried out with the aid of the Cystic Fibrosis Foundation Patient Registry (CFFPR) from 2017 to 2021 centered on newborn infants and found that CF patients have a median lifespan of ~53 years (Taylor-Cousar et al., 2023). In individuals with CF, the presence of two faulty CF genes and malfunctioning elements of the genetic machinery can result in multiplied stages of mucus in diverse organs, which can cause similar complications and impair the regular functioning of these organs.

This situation could have detrimental effects on the lungs, pancreas, liver, intestines, and salivary glands. On the other hand, although the CFTR channel and chloride ions are not functioning properly due to insufficient shipping, it causes the production of thick mucus. Mucous production obstructs the airways, making it hard to breathe and increasing the probability of bacterial lung infections in affected individuals (Blevings et al., 2022).

## Immune responses in cystic fibrosis

CF patients affected with COVID-19 show an exaggerated inflammatory reaction characterized by the release of positive chemical compounds and cells such as IL-8, TNF, mucin, polymorphonuclear leukocytes (PMNs), and serine proteases. These robust inflammatory reactions contribute to the further development of CF symptoms. The abnormal innate immune responses found in CF patients may be attributed to the CFTR mutation, which negatively affects the function of the epithelial innate immune system.

Under these circumstances, the NF-κB pathway, which induces activation of the positive genes, becomes active, resulting in a multiplied secretion of IL-8. The law of TLR4 and IFN-γ expression and accumulation is compromised in a weakened immune system. Additionally, the activation of pulmonary dendritic cells (DCs), which play a crucial role in T-cell-established immunity and immune surveillance, is decreased (Lambrecht et al., 2001).

## Epidemiology and clinical outcomes of COVID-19

Among the extremely good human coronaviruses (HCoVs), the novel coronavirus (SARS-CoV-2) is significant. HCoVs, which are RNA-enveloped viruses, can be transmitted from animals, such as rodent and bat families, to human beings. In December 2019, the World Health Organization (WHO) identified SARS-CoV-2 in Wuhan, China, ultimately leading to the prevalence of acute respiration distress syndrome (ARDS). On 11 March 2020, the novel coronavirus, known as COVID-19, was declared an international pandemic. It is crucial to highlight that SARS-CoV-2 is a newly diagnosed strain that has not been previously detected in humans. The outbreak ended with over 381,000 individuals being infected across 195 countries, with the number of deaths exceeding 16,000. In patients having inflammation with severe acute respiration syndrome coronavirus 2 (SARS-CoV-2), common signs and symptoms include low-grade fever, fatigue, dry cough, sore throat, diarrhea, anosmia, and loss of flavor or scent. While many people with this contamination might also be asymptomatic or experience slightly higher breathing tract symptoms, there are times when they will develop intense and acute respiratory distress syndrome (ARDS; Chams et al., 2020; Chen et al., 2020). Contracting COVID-19 can bring about numerous organ-related troubles, such as acute kidney injury, vascular blood clots, endothelial cell damage, and shock. These issues and headaches can substantially increase pressure on society and healthcare systems (Bradley et al., 2020; Chams et al., 2020; Yohannes, 2021).

## Microbiome changes linked to COVID-19

The microbiome consists of a wide variety of microorganisms, including bacteria, fungi, viruses, and protozoans, that live in distinct organs at some point in the human body. These microorganisms have a substantial function in regulating cell metabolisms and biological signaling pathways (Gilbert et al., 2018). Evidence supports the critical involvement of the microbiome community, especially intestinal microbiota, in maintaining a properly functioning bodily system, immune responses, and metabolic functions. In cystic fibrosis patients, there is regular dysbiosis of the intestinal microbiota over time, characterized by a reduction in the abundance of useful bacteria and an increase in pathogens. This imbalance can bring about inflammation and compromise immune responses. However, the impact of this dysbiosis on the susceptibility to or transmission of COVID-19 in these patients remains inadequately explored. Subsequent medical evaluations have found the prevalence of intestinal dysbiosis and changes in the respiration microbiome in people affected by COVID-19 (Segal et al., 2020). An observation in 2022 potentiated that the depletion of *Faecalibacterium prausnitzii* ought to contribute to respiratory and lung disorders along with allergies and cystic fibrosis (Vernocchi et al., 2018; Demirci et al., 2019). *F. prausnitzii* is believed to possess anti-inflammatory properties, protecting against various gastrointestinal illnesses, including Crohn's disease (Parada Venegas et al., 2019; Leylabadlo et al., 2020). Furthermore, more recent research on COVID-19-recovered patients with persistent symptoms discovered that post-exercising chest tightness becomes inversely associated with the relative abundance of *F. prausnitzii* (Zhou et al., 2021). Throughout the duration of hospitalization in Hong Kong, the stool samples of the 15 COVID-19 patients displayed the growth of opportunistic bacteria such as *Rothia, Streptococcus, Actinomyces*, and *Veillonella*, as indicated by similar examinations. Additionally, a lower bacterial range was found in these samples (Gu et al., 2020). Moreover, alterations in the gut and lung microbiomes may facilitate the infiltration of the SARS-CoV-2 virus into lung tissue. The lung microbiomes play critical roles in the onset, development, and efficacy of therapeutic interventions (Ye et al., 2020).

## Entry of SARS-CoV-2 of SARSCoV-2; outcome in CF patients

The SARS-CoV-2 virus enters cells by using the spike (S) protein, which is located on its surface. This protein is made up of two parts, S1 and S2. The S1 subunit attaches to the angiotensin-converting enzyme 2 (ACE2), which is the main receptor on the surface of certain cells in the airway epithelium. These cells include airway epithelial cells, goblet secretory cells, and type II cells during pneumocystis infection. When the virus enters a host cell, the S1 domain of the S protein binds to ACE2, causing the S1 subunit to be cut by cellular proteases. Then, the S2 subunit helps the viral membrane fuse with the host cell membrane, allowing viral components to be released into the host cell's cytoplasm (Shirato et al., 2017; Chams et al., 2020; Touret et al., 2020; Zhao et al., 2022).

**Figure 1:**
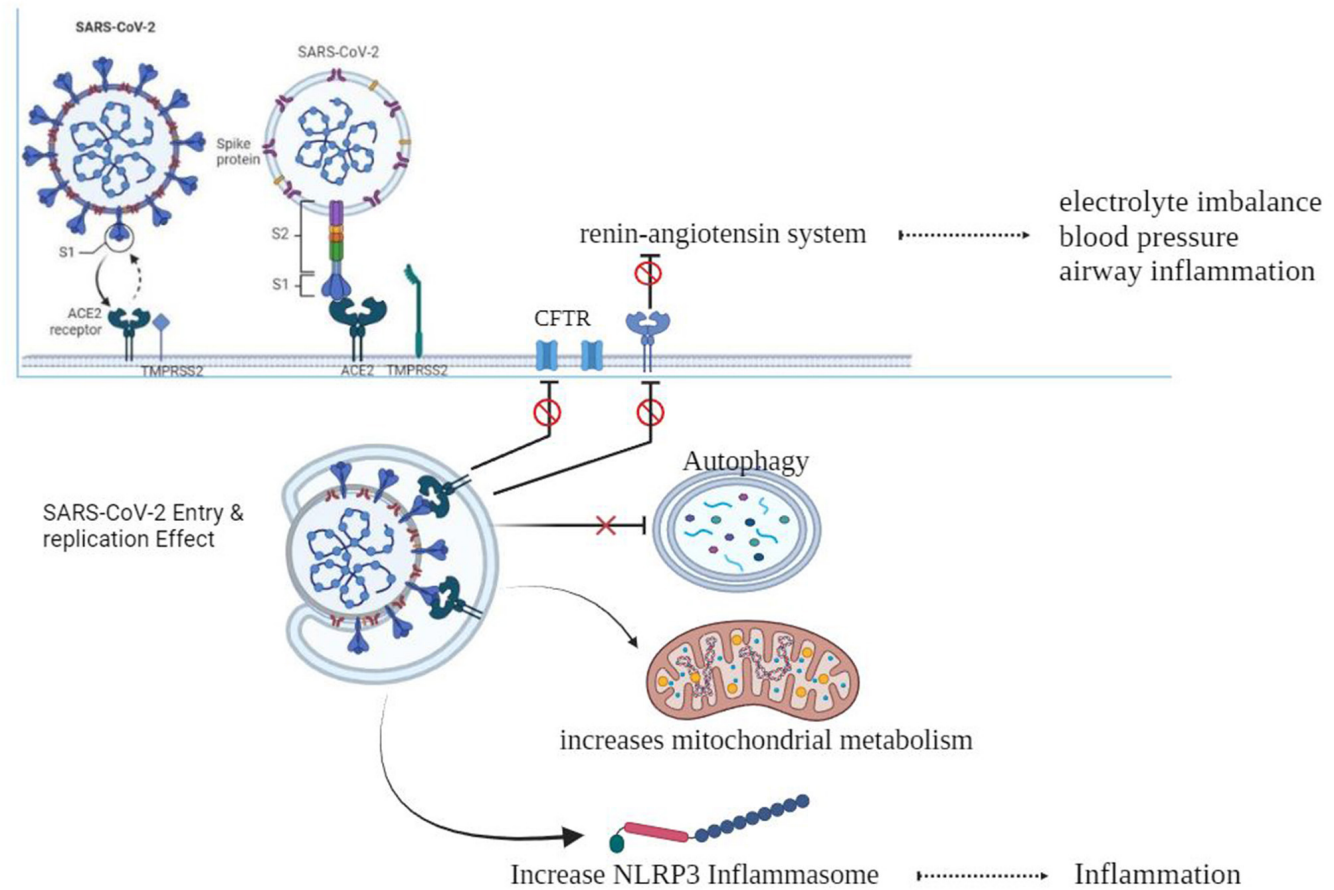
SARS-CoV-2 and cystic fibrosis.

As shown in Figure 1, When the spike protein attaches to the ACE2 cell membrane protein, an association is formed between the virus and the cell. The enzymes TMPRSS2 and furin help in the entry of the virus into the cell, and individuals with CF may have altered versions of these enzymes (Chams et al., 2020; Peckham et al., 2020). There is a z report of a decreased level of TMPRSS2 among CF patients compared to the control group, although it was not significant, and there is also an increase in the TMPRSS2 enzyme in CF patients by flagellin of *Pseudomonas aeruginosa* (Bitossi et al., 2021; Ruffin et al., 2021).

After entering the cell, the virus can be affected by excessive inflammation and CF-related cellular processes like autophagy, mitophagy, endosomal function, and cellular metabolism. SARS-CoV-2 may take advantage of these cellular processes to replicate itself (Peckham et al., 2020).

CF can result in electrolyte abnormalities, while the SARS-CoV-2 infection hampers the functioning of pulmonary ACE2, resulting in the disruption of the renin–angiotensin system (RAS). This disruption has detrimental outcomes on fluid and electrolyte balance, blood pressure, and airway irritation. Furthermore, it additionally complements vascular permeability in the airways, as indicated in previous studies (Scurati-Manzoni et al., 2014; Bekassy et al., 2021).

## Replication of SARSCoV-2; outcome in CF patients

**Figure 2:**
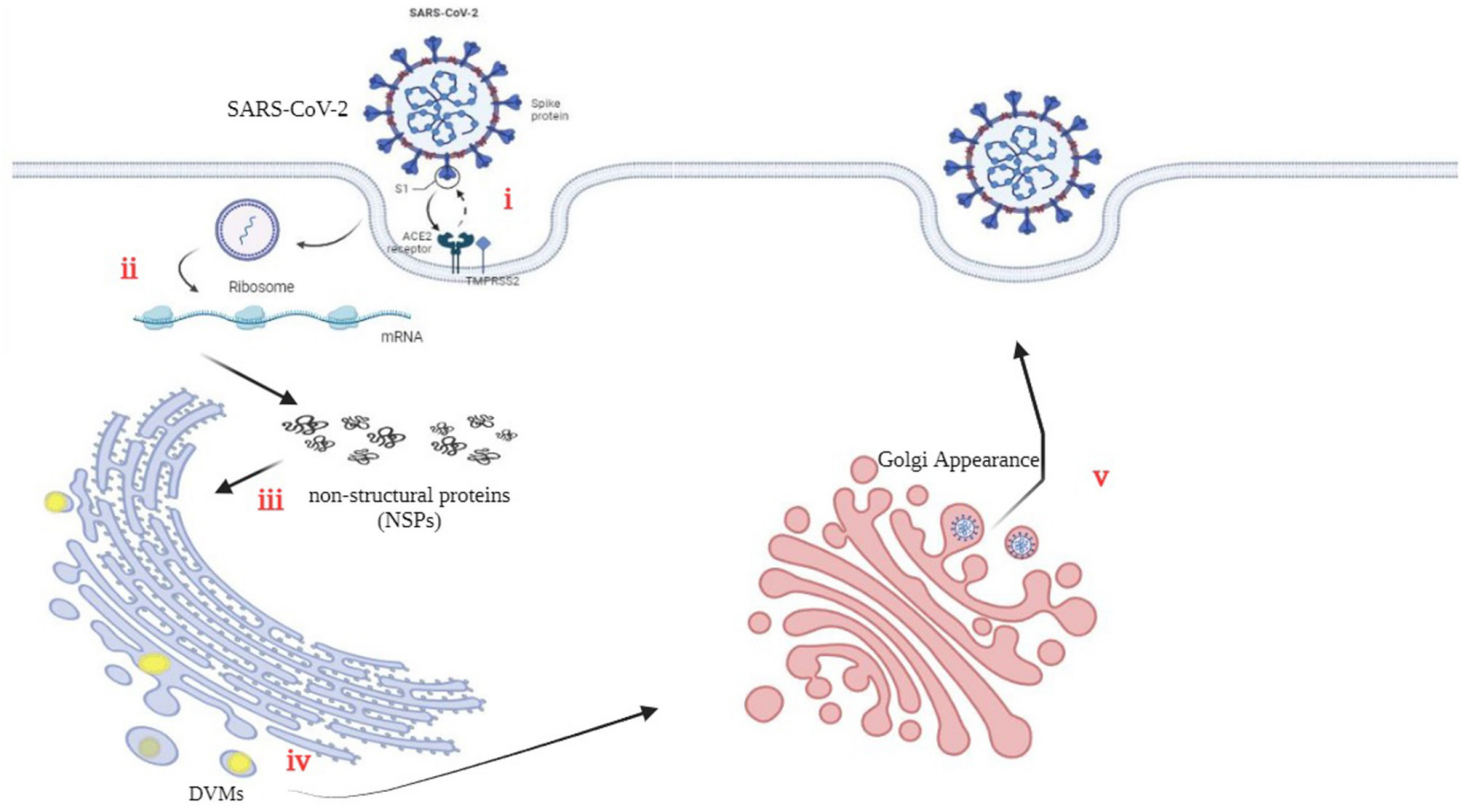
An illustrative representation of the replication cycle in SARS-CoV-2.

The three-chymotrypsin-like cysteine protease (3CLpro) of SARSCoV-2 is a vital component of the virus that plays a key function in virus replication and is conserved. This protease cleaves the polyproteins to generate 16 useful non-structural proteins (NSPs). The cleaved NSPs have essential functions in assembly and replication (Mody et al., 2021). Figure 2 illustrates the precise interaction between viral S-glycoproteins and the cell ACE2 receptor, which allows the entry of SARS-CoV-2 into cells. The spike glycoprotein is cleaved through TMPRSS2, which helps fusion among the host cellular membrane and the virus envelope (Hoffmann et al., 2020). Viral RNA is translated into non-structural proteins (NSPs). (iii) Furthermore, the virus utilizes the host cellular machinery to translate viral proteins. The RNA genome is replicated and translated into double-membrane vesicles (DMVs), which resemble bubble-like endoplasmic structures. (v) Finally, the mature virion is transported to the Golgi bodies, where it is launched through exocytosis (Yuan et al., 2020; Roingeard et al., 2022).

The availability of the CFTR protein for the SARS-CoV 3CL protease may result in its cleavage (Peckham et al., 2020). Additionally, evidence suggests that the airway tract microbiota, thick secretions, and autophagy induction in CF patients may play a protective role against viral infections. Autophagy, as an immune mechanism in CF disease, could be heightened and actively contributes to the antiviral response (van Ewijk et al., 2005; Junkins et al., 2014; Mingione et al., 2020; Pehote and Vij, 2020).

## Immune system status in patients affected with COVID-19 simultaneously with CF

At the onset of contamination, SARS-CoV-2 penetrates host cells, permitting its genome to enter the cytoplasm and initiate a pro-inflammatory reaction via diverse signaling pathways. During the preliminary levels of viral infection in airway epithelial cells, regulated and programmed cell death responses known as pyroptosis take place, releasing seasoned-inflammatory cytokines and chemokines such as IL-6, IFNγ, MCP1, and IP-10. In the presence of inflammatory cytokines, immune cells are recruited to eliminate host epithelial cells, thereby diminishing inflammatory immune responses in the lungs. Nevertheless, in SARS-COV-2 positive patients, induction of a cytokine storm, a deadly immune system reaction that may damage multiple organs, including the coronary heart, liver, and kidneys, in the long run may result in organ failure (Tay et al., 2020; Yapasert et al., 2021).

The innate immune system plays an essential role in the preliminary defense in opposition to COVID-19 (Diamond and Kanneganti, 2022). Among the various cytokines being considered for the improvement of COVID-19, interleukin-6 (IL-6) often emerges as the most significant. Elevated levels of IL-6 are directly associated with the development and mortality rates of this viral infection, which may be triggered by the initiation of SARS-CoV-2. Importantly, this research shows that lowering IL-6 levels ought to serve as a protective measure against SARS-CoV-2 infection, specifically in the context of cytokine storms (Chams et al., 2020; Coomes and Haghbayan, 2020; McGonagle et al., 2020). In a comprehensive study involving 39 CF patients with persistent pulmonary infections, an evaluation of accumulated sputum found a decrease in IL-6 and interleukin-10 (IL-10) levels, followed by an increase in interleukin-8 (IL-8) levels. The production of IL-6 within localized sputum was found to decrease, even as systemic IL-6 levels remained unaffected (Majka et al., 2021).

The airway tract of people with cystic fibrosis suffering lung damage indicates an increased concentration of neutrophil elastase, a dangerous pathogen, leading to a decrease in pulmonary function and activities. The incidence of pulmonary infections, together with acute respiratory distress syndrome (ARDS), related to COVID-19, has been linked to the increased secretion of excess neutrophil elastase. According to available evidence, the capacity treatment (therapy, cure, medication, repair, solution, medicine, fix, correct, solutions, solve, prevent, remedies, recourse) for lung damage and ARDS includes the use of neutrophil elastase inhibitors (Mohamed et al., 2020; Sahebnasagh et al., 2020; Yang and Montgomery, 2021; Terlizzi et al., 2022a,b). Nebulized dornase alfa, which breaks down DNA and promotes the discharge of extracellular traps of neutrophils, has been identified as having a useful function in the development of lung disease. Its use has shown promise in improving lung characteristics in cystic fibrosis patients and can be taken into consideration for trials for COVID-19 treatment. Although the current research proposes against the persistent use of nebulized dornase alfa, the administration of azithromycin has been indicated as an effective remedy for COVID-19 in some research (Kournoutou and Dinos, 2022).

## Epidemiological and clinical outcomes of SARS-CoV-2 in CF patients

Recent findings propose that the majority of children infected with the quite contagious SARS-CoV-2 virus exhibit mild signs of inflammation. However, it remains uncertain whether children with continual respiratory symptoms experience exacerbated symptoms due to SARS-CoV-2. Nevertheless, initial studies suggest that most children infected with SARS-CoV-2 exhibit mild signs and symptoms (Borch et al., 2022). The recent findings revealed that children recognized with bronchial asthma and cystic fibrosis, who have reduced weight and contracted SARS-CoV-2, did not display a considerable decrease in lung characteristics. This finding is especially important as many children with bronchopulmonary dysplasia (BPD) and other breathing disorders require ventilator assistance, making them more prone to infections inside this group (Ward, 2016; Kaore and Kaore, 2021). A study carried out across more than one country, together with the United States, verified that the duration of infection in people with CF corresponds to that of the general population. However, due to the small sample size of the studies, definitive and particular conclusions were hard to draw. Therefore, it is widely suggested that people with CF adhere strictly to public health policies to safeguard themselves from infections (Jin et al., 2020). In cases in which people are recognized as having cystic fibrosis (CF) and sooner or later are identified as having SARS-CoV-2, the severity of infection can be further intensified, potentially leading to an escalation in signs and symptoms (Hong et al., 2020). Several investigations were conducted in this specific context. In February 2020, a total of 13 individuals recognized with cystic fibrosis (CF) were found to be suffering from COVID-19 (Fainardi et al., 2020).

The COVID-19 outbreak in Northern Italy had a substantial effect on CF patients, especially those living in areas with an excessive occurrence of the ailment. It is known that every CF patient who contracted the virus had been infected through their circle of relatives, such as close people or related people. Among those patients, 61.5% experienced slight breathing difficulties; at the same time, the remaining 38.5% required more intensive clinical interventions. Furthermore, out of the 13 patients, COVID-19 was detected in others as well. However, in May 2020, a significant number of patients, especially 85%, had recovered (Fainardi et al., 2020).

In contrast to European nations with similar populations, the areas in northern Italy, namely, Lombardia, Emilia-Romagna, Veneto, and Piemont, witnessed a substantially higher mortality rate due to the referred infection.

COVID-19 has been observed to have an excessive effect on the elderly population, with men being more vulnerable to infection than women. Consequently, a considerable number of elderly individuals in Italy have a higher mortality rate (Salzberger et al., 2021). Moreover, people with underlying health conditions, which include diabetes, cardiovascular diseases, or most cancers, are at a higher risk of death than those without these health conditions. Recent studies have additionally suggested cases of COVID-19 infections in kids (Mehrabani, 2020). Notably, the general population tested for an additional occurrence of SARS-CoV-2 (0.15%) in comparison to CF patients (0.07%; Cosgriff et al., 2020). This unexpected discovery could potentially be ascribed to the relatively younger age of patients affected with CF. The utilization of antibiotic treatment among CF patients confers protection against SARS-CoV-2.

CF patients also take precautions to prevent the spread of infection, including self-isolation, which probably contributes to the low occurrence of SARS-CoV-2 infection in this population (Bezzerri et al., 2020; Biondo et al., 2022). The Cystic Fibrosis Center in Parma endorsed self-isolation measures, including hand hygiene, sporting face masks, and remote verbal exchange, to reveal and avoid group activities throughout the pandemic period in Italy. Based on the results, it could be concluded that individuals suffering from extreme respiration infection as a result of the coronavirus may be correctly managed and guarded (Fainardi et al., 2020).

In an examination conducted in France, the findings imply that patients with excessive lung ailments resulting from COVID-19 may be affected. A study conducted in France determined that, out of 31 CF patients who have mild COVID-19, only 0.41% of them had inflammation, which resulted in a significant decrease (93% less) compared to the total number of members (Corvol et al., 2020). The study also found that CF patients with COVID-19 had a higher average age than the general population (Mathew et al., 2021), suggesting a correlation between age and coinfection with the virus. Symptoms such as fever, fatigue, and aggravated cough were mentioned in 28 patients, while the other three patients did not display any signs (Corvol et al., 2020).

Another study in Spain, at some stage in the peak of the preliminary wave of the pandemic, used RT-qPCR to confirm the presence of COVID-19 in CF patients (Mondejar-Lopez et al., 2020). CF patients with COVID-19 had a higher likelihood of being hospitalized as compared to the general population. Moreover, people with CF are more likely to experience worsening of their persistent lung ailment, which may lead to respiratory tract viral infections. Research has shown that CF patients have a lower prevalence of COVID-19 infection than the overall population (Mondejar-Lopez et al., 2020).

## Cystic fibrosis treatment: outcome in SARS-CoV-2 infection

Prolonged usage of antibiotics, such as azithromycin, acknowledged for its anti-inflammatory properties, can suppress viral infections in CF patients. Moreover, this antibiotic reveals antiviral consequences in a model of continuous obstructive pulmonary disorder (COPD; Khezri et al., 2021; Suarez-Reyes and Villegas-Valverde, 2021). Azithromycin, an often prescribed antibiotic for cystic fibrosis (CF), holds promise for decreasing the severity of COVID-19 because of its potential to modulate the immune response and show slight antiviral properties (Echeverria-Esnal et al., 2020; Ghazy et al., 2020; Touret et al., 2020). Furthermore, mutations in the CFTR gene may impact the performance of ACE2 and TMPRSS2 proteins, resulting in reduced susceptibility to SARS-CoV-2 infection (Stanton et al., 2020).

The significance of microorganisms, mainly *Pseudomonas aeruginosa* and *Staphylococcus aureus*, in the treatment of continual and acute infections is noteworthy. When infection arises inside the lungs, it leads to pulmonary exacerbation (PEx), inflicting a decline in lung characteristics and negatively affecting normal, excellent lifestyles (Lam et al., 2015).

Previous research has indicated that bacterial infections can affect immune responses to viral infections (Nilashi et al., 2020). A previous study found that viral infections currently do not have a sizeable impact on lung features in patients with CF (Smith et al., 1980). In fact, it can be asserted that the suppression of bacterial colonization, in particular *Pseudomonas aeruginosa* as formerly stated, is essential in dealing with CF infection. The presence of viral microorganisms played a considerable position in the exacerbation of CF (Wark et al., 2012).

Medications and specialized treatments administered to people with CF can relieve the severity of COVID-19. It is plausible that pills used in the treatment of CF sufferers may be connected to a reduction in COVID-19 symptoms, playing an important function in mitigating the severity of the disorder (Gaudio, 2020; Porter et al., 2023).

## Effect of host factors of CF patients on SARS-CoV-2 infection

The variation in COVID-19 and mortality rates among distinct countries, in conjunction with the various clinical manifestations of the viral infection in patients, has been drastically documented (Sorci et al., 2020). Host genetic elements were recognized as vital elements influencing the pathogenicity of COVID-19 (Tharappel et al., 2020; Jafarpour et al., 2021). Specifically, individuals carrying single pathogenic versions of the CFTR gene (CF vendors) are more susceptible to respiratory tract infections and severe COVID-19 (Baldassarri et al., 2021). ACE polymorphisms have additionally been related to COVID-19, as studies have indicated that patients with the ACE D/D polymorphism showcase advanced medical signs and symptoms and a higher danger of lung damage in comparison to people with I/I or D/I polymorphisms (Karakaş Çelik et al., 2021). Furthermore, studies have validated that ACE polymorphisms can affect ACE2 expression, leading to the CF phenotype and pulmonary inflammation associated with the development of COVID-19 (Vitiello et al., 2023). It is critical to highlight that the angiotensin-converting enzyme 2 (ACE2) serves as the crucial host receptor for SARS-CoV-2 entry via the spike (S) protein on the virus surface (Zhang et al., 2020).

## Impact of COVID-19 on individuals with cystic fibrosis

To gain a complete knowledge of the effect of COVID-19 on individuals with cystic fibrosis, it is essential to accumulate additional proof and records. The European Cystic Fibrosis Society (ECFS) performs an important role in this regard by gathering proof statistics and disseminating well-timed and vital documents from various areas across Europe (Colombo et al., 2020). Respiratory infections affecting the respiratory system are more intense in cystic fibrosis patients than the overall population (Yu and Kotsimbos, 2023). Furthermore, complications and destructive effects on lung characteristics were determined to be increasing among individuals with cystic fibrosis (Flume et al., 2019). While a few individuals with cystic fibrosis may additionally experience respiratory fitness troubles, others may also be afflicted by a chronic airway ailment characterized by intense signs and symptoms (Hisert et al., 2017). It is crucial to note that the signs of cystic fibrosis differ significantly from the clinical manifestations of COVID-19 (Al Lawati et al., 2023).

Investigations have confirmed that organ transplantation is a determinant of COVID-19 prevalence. Consequently, it has been found that lung transplant recipients with COVID-19 infection have a higher mortality rate than those note affected with COVID-19 (Pereira et al., 2020; Hall et al., 2022). In patients with cystic fibrosis (CF), mainly those experiencing extreme respiratory infections, it is important that we no longer miss the possibility of COVID-19 contamination because of reduced immunity. Despite the incredibly low prevalence of SARS-CoV-2 infections in CF people, it is more important to impress upon this population the importance of using numerous measures together with effective somatic and public distancing, as well as using structures and practices aimed toward infection management. These measures should be incorporated into ordinary CF care. Additionally, different elements can also make a contribution to the management of COVID-19 in CF patients (Mathew et al., 2021).

Hence, it is more feasible for people with mild COVID-19 to be mistakenly diagnosed as having CF, while those with slight signs and symptoms can be perceived as healthy individuals. To tackle this trouble, a low-threshold trial is proposed to be implemented, which will facilitate early detection. Given the cutting-edge instances, a wide variety of households have expressed issues concerning the right of entry to medicinal drugs and food supplies (Burgel and Goss, 2021; Lim et al., 2022). To maintain excellent health, patients with cystic fibrosis and their families must adhere to certain principles. It has been observed that CF patients have been successful in averting infection with SARS-CoV-19 (Colombo et al., 2020). Researchers are presently accumulating various statistics to determine the elements that affect the severity of COVID-19 within the cystic fibrosis patients. With the spread of the SARS-CoV-2 pandemic, it is essential for us to accumulate diverse records to understand how this viral infection influences specific patient groups with unique illnesses, which includes cystic fibrosis (Carr et al., 2022). One study found that 181 cystic fibrosis patients (32 post-transplant) from 19 countries were infected with SARS-CoV, and infection with SARS-CoV-2 had similar effects as determined within the general population (McClenaghan et al., 2020). One study demonstrated that only a small number of individuals from the Cystic Fibrosis Registry had been diagnosed with or examined for COVID-19 on a month-to-month basis as of January 2020 (Berardis et al., 2020; Colombo et al., 2020; Corvol et al., 2020; Cosgriff et al., 2020; McClenaghan et al., 2020; Scagnolari et al., 2020; Bain et al., 2021; Naehrlich et al., 2021).

The association between scientific cycles and certain elements, which include older age, CF-associated diabetes, decreased lung features within the 12 months before contamination, and having gone through an organ transplant, has been observed (Jardel et al., 2018; Mainbourg et al., 2019). Despite the higher-than-anticipated effects in a large cohort, probably because of the noticeably younger CF population as compared to other continual situations, it is critical to say that SARS-CoV-2 is not a benign ailment for all individuals in this group of affected persons (Hadi et al., 2021). The COVID-19 global pandemic as a result of SARS-CoV-2 has had a huge impact and continues to unfold globally, with CF being identified as a potential factor for negative effects (Taylor-Cousar et al., 2023). Multiple national CF registries from diverse countries have documented a lower prevalence of SARS-CoV-2 contamination in people with cystic fibrosis (PwCF) in comparison to the general population (Cosgriff et al., 2020; Flume et al., 2022). Furthermore, it has been discovered that more young PwCF tend to have milder signs and symptoms and inflammation with SARS-CoV-2. However, individuals with compromised immune systems and people with impaired lung function are more likely to experience intense results (Burgel and Goss, 2021). Another study applied the multicenter research community TriNETX method to research the medical outcomes of COVID-19 contamination in a large cohort of PwCF in an evaluation of the general populace. The findings found that, out of the 507,810 individuals aged 6 years or older, women constituted the majority (*n* = 225, 53.32%), and the common age at COVID-19 analysis among CF patients was ~46.6 years. The majority of the participants were of Caucasian ethnicity (*n* = 309, 73.22%). Higher rates of hospitalization, acute renal damage, and critical care needs were found in PwCF following robust propensity matching.

Hospitalization became vital for 10% of patients with COVID-19 (Hadi et al., 2021). Despite the reality that CF patients have a decreased prevalence of obesity and a lesser median age, these elements no longer offer protection against intense disease. A prospective multicenter cohort study, which involved 32 CF centers and 6,597 patients, was conducted to study the symptoms and scientific path of SARS-CoV-2 infection in PwCF. It was found that dysfunctional kidneys, respiration systems, dying facts, and unrivaled reviews were also improved in PwCF at 30 days (Colombo et al., 2021). To ensure proper follow-up, facilities reached out to individuals showing symptoms indicative of COVID-19. According to a recent publication (Tedbury et al., 2023), CFTR may additionally affect the severity of SARS-CoV-2 infection and COVID-19 ailments in CF patients (Vitiello et al., 2023). Meanwhile, a literature evaluation (Marques et al., 2023) emphasizes the restricted understanding regarding the effects of COVID-19 on CF patients. The evaluation indicates that CF patients may be at a greater risk of intense infection from COVID-19 due to their underlying lung disease and other comorbidities (Marques et al., 2023). Additionally, an examination carried out in Brazil indicates using observational studies to assess the results of COVID-19 on individuals diagnosed with cystic fibrosis (Table 1; Sorci et al., 2020).

**Table 1:** Studies that have been done in the field of COVID-19 infection in CF patients.

				**COVID-19 in CF**				
**References**	**Study type**	**Study population**	**Study period**	**Diagnostic method**	**Sample size**	**Positive cases**	**Incidence**	**Summary**
Mondejar-Lopez et al. (2020)	Retrospective descriptive observational	Spanish CF patients	8 March−16 May 2020		39	8	3.2/1,000	Low incidence and mortality in CF patients vs. the general population.
Berardis et al. (2020)	Monocentric prospective study	Belgian CF patients	16 April−19 May 2020	Anti-SARS-CoV-2 IgM and IgG antibodies	149	4	-	Difficulty in distinguishing COVID-19 symptoms from respiratory exacerbations.
Naehrlich et al. (2021)	Observational	16-country of Europe	01 February−30 June 2020	PCR	48,148	130	2.7/1,000	SARS-CoV-2 infection can result in severe illness and death for pwCF, even for younger patients.
McClenaghan et al. (2020)	Experimental	19 countries	Up to the 13 of June 2020	PCR and/or CT scan,	85,000	181	2.1/1,000	SARS-CoV-2 is not a benign disease for all people in the CF population.
Peckham et al. (2020)	Brief communications	-	-	-	-	-	-	Overlaps of cytokine dysfunction and hyper-inflammation in the pathophysiology of COVID-19 and CF patients
Jung et al. (2021)	Prospective observational	26 countries	Up to December 31, 2020	PCR	48,211	828	17.2/1,000	Increased risk of severe outcomes in PwCF with CFRD and those with lung transplants.
Mathew et al. (2021)	Systematic review	30 countries	April 28 and December 10, 2020.	-	-	339	-	It is recommended by published evidence today which is important to the monitoring of the risk of COVID-19 in some groups of PwCF.
Fainardi et al. (2020)	Review	Italy	Up to 3 August 2020.	-	-	-	-	Worldwide, a small number of cystic fibrosis patients, mostly adults, are contracting SARS-CoV-2, with no noticeable impact on the severity of their cystic fibrosis. The findings indicate that patients with cystic fibrosis may have some form of protection against severe lung disease caused by SARS-CoV-2.
Stanton et al. (2020)	Review	Germany	Up to 4 July 2020.	-	-	-	-	Ecotin, SERPINB1, camostat mesylate, nelfinavir mesylate, chloromethyl ketone, azithromycin, and ciprofloxacin are factors that could reduce the severity of SARS-CoV-2 in CF patients.
Colombo et al. (2022)	Prospective	Italian CF centers	Between March 2020 and June 2021	PCR	-	236	-	COVID-19 should not be regarded as a minor illness in individuals with cystic fibrosis, especially those with significantly compromised respiratory function and organ transplants.
Baldassarri et al. (2021)	Cohort	Italian CF centers	8 April to 30 June 2020	PCR	874	40	-	It is important to examine the status of cystic fibrosis carriers in COVID-19 hospitalized patients because of its high occurrence. This will help identify individuals at risk of severe illness who could benefit from close monitoring and tailored treatment.
Terlizzi et al. (2022c)	Systematic review	-	1st of January 2020 to the 6th of November 2021	-	-	-	-	While most individuals with CF have a benign course of SARS-CoV-2 infection, several subgroups at higher risk of catastrophic outcomes have been identified.
Marques et al. (2023)	Systematic review	-	17 November 2019–23 May 2022	-			-	PwCF may face a higher risk of COVID-19 than the general population due to the disease and public health measures such social isolation, which can limit their capacity to exercise.
Bhatnagar et al. (2023)	Original article	Ireland CF patients	-	-	119	16	-	The COVID-19 pandemic has significantly affected PwCF in terms of hospital visits, access to diagnostics, cystic fibrosis care, and psychological wellbeing. Youth with cystic fibrosis expressed a more significant effect on their mental wellbeing.

CF, cystic fibrosis; SARS-CoV-2, severe acute respiratory syndrome coronavirus 2; pwCF, people with CF; CFRD, cystic fibrosis-related diabetes; *SARI*, severe acute respiratory infection; PCR, polymerase chain reaction.

## Conclusion

Various breathing infections have posed a hazard to individuals with CF, regularly leading to excessive consequences. However, the contribution of viral infections to CF pulmonary decline remains a subject of research. Research suggests that CF patients are prone to acute breathing viral infections, yet it appears that most people have not been critically laid low with SARS-CoV-2. Conversely, reports advise that CF patients who have undergone transplantation and finally got mild infections have been laid low with the severe SARS-CoV-2 strain. Another cause of the particularly low occurrence of this virus among CF patients is the commonly lower age of this populace. Given that many CF patients are young people and have constrained receptors for the SARS-CoV-2 virus, the occurrence and severity of the ailment are generally decreasing in this group. Recent research has proposed numerous protective mechanisms for SARS-CoV-2 infection in CF patients. These investigations have highlighted the complete function of CFTR in SARS-CoV-2 replication, as CFTR deficiency results in reduced viral replication. Panou (2018) and Lotti et al. (2022) have said that inhibiting CFTR in primary kidney cells inflamed with BK polyomavirus (BKPyV) drastically reduces the transportation of virions to the ER. Recent studies have indicated that impaired CFTR features may also have a huge effect on viral replication. Specifically, the activation of the SARS-CoV-2 S protein depends on endolysosomal proteases in acidification, and deacidification of this organelle has been shown to prevent viral infection. Mutations in the CFTR gene can result in an increase in organelle pH, resulting in altered glycosylation patterns of ACE-2 and/or TMPRSS-2, which could affect the results of SARS-CoV-2 infection. Furthermore, modifications in ionic balance can modify intracellular pH, leading to large modifications in viral protein assembly and structure (Stanton et al., 2020).

From a pathogenic perspective, increased stages of neutrophil elastase are associated with extended lung damage and reduced pulmonary characteristics in CF (Dittrich et al., 2018; Barth et al., 2020). Consequently, neutrophil elastase inhibitors are being actively examined in trials for CF treatments (Barth et al., 2020). It is worth noting that an imbalance of seasoned-inflammatory neutrophil elastase is also implicated in the improvement of acute respiratory distress syndrome (ARDS) associated with COVID-19 (Polverino et al., 2017). Therefore, neutrophil elastase inhibitors were proposed as capacity-repurposed treatments for ARDS and the associated lung damage (Sahebnasagh et al., 2020).

Additionally, nebulized dornase alfa, a typically used CF treatment, is presently undergoing trials for COVID-19 treatment (Southern et al., 2019; Earhart et al., 2020). Its proposed protective impact is attributed to its clearance of neutrophil extracellular traps, which play a pathogenic function in SARS-CoV-2 infection (Okur et al., 2020). Interestingly, preliminary studies show that dornase alfa is effective in proscribing the *in vitro* contamination of green monkey and bovine kidney cell strains with the aid of SARS-CoV-2 (Okur et al., 2020).

In conclusion, azithromycin, another usually prescribed antimicrobial agent, has been proven to have antiviral effects against SARS-CoV-2. In the preliminary research, the use and effectiveness of nebulized dornase alfa have not yet been truly defined, but the use of azithromycin was referred to in four research studies (Corvol et al., 2020; Moeller et al., 2020; Mondejar-Lopez et al., 2020; Bain et al., 2021), both as a pre-present treatment or as a capability treatment for COVID-19. It is possible that CF patients exposed to SARS-CoV-2 can be on medications that could alleviate the severity of COVID-19.

Overall, the studies referred to in this evaluation advise that different factors might also help lessen the severity of SARS-CoV-2 in CF sufferers and identify potential goals for further studies to mitigate the severity of SARS-CoV-2 in both CF patients and the general population. These factors encompass ecotin, SERPINB1, camostat mesylate, nelfinavir mesylate, chloromethyl ketone, azithromycin, and ciprofloxacin, some of which are presently being tested in scientific trials (Stanton et al., 2020). Additionally, the composition of the lung microbiome in CF patients, which includes opportunistic pathogens, non-tuberculosis mycobacteria (NTM), and fungi, may play a role in the functioning of COVID-19. Further research in those areas ought to offer valuable insights and future prospects for investigation.

While the findings advocate that CF patients can be truly covered in opposition to extreme lung disorders resulting from SARS-CoV-2, the medium and long-term effects of SARS-CoV-2 on infected CF patients remain unknown. Further research on a larger population of CF patients is needed to determine the proper impact of SARS-CoV-2 on CF lung ailment. Additionally, given the different factors contributing to the spread of SARS-CoV-2, it is encouraged that CF patients be closely monitored to shield them from the risk of COVID-19 contamination.

## Author contributions

FA: Data curation, Software, Writing – original draft. NR: Data curation, Writing – original draft. HA: Conceptualization, Supervision, Writing – original draft. PS: Writing – review & editing. MS: Project administration, Supervision, Validation, Writing – review & editing. MMod: Writing – review & editing, Visualization. MMoe: Data curation, Conceptualization, investigation, Software, Writing – review & editing.
